# Viruses, cancer and non-self recognition

**DOI:** 10.1098/rsob.200348

**Published:** 2021-03-31

**Authors:** Monikaben Padariya, Umesh Kalathiya, Sara Mikac, Katarzyna Dziubek, Maria C. Tovar Fernandez, Ewa Sroka, Robin Fahraeus, Alicja Sznarkowska

**Affiliations:** ^1^ International Centre for Cancer Vaccine Science, University of Gdansk, Kladki 24, 80-822 Gdansk, Poland; ^2^ Inserm UMRS1131, Institut de Génétique Moléculaire, Université Paris 7, Hôpital St. Louis, F-75010 Paris, France; ^3^ RECAMO, Masaryk Memorial Cancer Institute, Zluty kopec 7, 65653 Brno, Czech Republic; ^4^ Department of Medical Biosciences, Umeå University, Building 6M, 901 85 Umeå, Sweden

**Keywords:** major histocompatibility (MHC) class I, virus–host interactions, viral immune evasion, cancer immune evasion, viral persistence

## Abstract

Virus–host interactions form an essential part of every aspect of life, and this review is aimed at looking at the balance between the host and persistent viruses with a focus on the immune system. The virus–host interaction is like a cat-and-mouse game and viruses have developed ingenious mechanisms to manipulate cellular pathways, most notably the major histocompatibility (MHC) class I pathway, to reside within infected cell while evading detection and destruction by the immune system. However, some of the signals sensing and responding to viral infection are derived from viruses and the fact that certain viruses can prevent the infection of others, highlights a more complex coexistence between the host and the viral microbiota. Viral immune evasion strategies also illustrate that processes whereby cells detect and present non-self genetic material to the immune system are interlinked with other cellular pathways. Immune evasion is a target also for cancer cells and a more detailed look at the interfaces between viral factors and components of the MHC class I peptide-loading complex indicates that these interfaces are also targets for cancer mutations. In terms of the immune checkpoint, however, viral and cancer strategies appear different.

## Graphical abstract

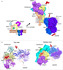

## 1. Introduction

Viruses have been, and still are, an invaluable source for understanding various aspects of cell biology. The Rous sarcoma virus revealed viral oncogenesis while simian, human adeno (HAdV) and human papilloma (HPV) viruses paved the way for the identification of the p53 and the Retinoblastoma protein (pRB) tumour suppressors. Similarly, viruses have played an important role in our understanding of how the immune system distinguishes self from non-self, both in terms of how the innate response detects pathogen patterns and how the adaptive immune system specifically detects the presence of neoantigens on the major histocompatibility class (MHC) molecules. Different strategies employed by viruses to evade the immune system reflect the balance of specific virus–host interactions and point out distinct steps in antigen presentation and the strategies best serving each virus. Virus–host interactions are commonly seen as good versus bad, but this is an oversimplification. In fact, more recent works illustrate how viruses have adapted to the hosts' immune system and how they have helped to create it, not only as a consequence but also as a cause, pointing towards a more balanced picture that reflects the coevolutionary origin of viruses and their hosts [1–4].

There are several mechanisms in place to help the immune system distinguish between self and non-self. The detection of pathogen-associated molecules by innate cells such as dendritic (DC) macrophages results in the release of inflammatory cytokines and interferons that promote the activation of the adaptive response or, in the case of NK cells, the elimination of stressed cells. The DCs play an important role in the interface between the innate and the adaptive immune system by presenting antigens on their MHC class I and II molecules that helps activate CD4^+^ and CD8^+^ T cells. Specific T cell receptors (TCR) on CD8^+^ T cells detect 8–10 amino acids peptides presented on MHC class I molecules and form a cornerstone in the immune system's capacity to distinguish self from non-self and for the elimination of virus-infected, damaged or transformed cells. Consequently, the evasion of the MHC class I pathway plays a major role in persistent viral strategies to minimize the risk of the infected host cell being detected and removed by the immune system. Similarly, cancer cells expressing neoantigens also need to evade the immune system and use similar—but also (as we will describe) different—strategies. However, the underlying processes of how cells select antigens to be presented on MHC class I molecules are still not fully understood, and a main challenge for the immune system to stay sensitive to pathogen infections or to detect mutated genes is the fact that the number of MHC class I molecules are very few compared to the number of peptides produced [5–7]. Also, each T cell expresses one TCR and the number of T cells is much lower than the number of potential antigenic peptides [8,9]. How the immune system ‘deals’ with this and stays sensitive to non-self is not yet fully understood.

Our limited understanding of virus–host coevolution results from the perspective that viruses are mainly disease-causing agents (run-away acute replicators) and the successful host escapes from the disease, resulting in a constant ‘arms race’ between viruses (predator) and their hosts (prey). However, acute replication is not the predominant lifestyle of viruses, nor is it conserved on the long-term scale, but mainly serves to explore new host possibilities [1,2,10]. The predominant successful and evolutionarily conserved viral strategy is persistence, which is the capacity of a functional or defective virus to colonize an individual host and be maintained or re-emerge at some later time [2]. It results in an intimate relationship between the virus and the host that relies on the coordinated expression of the virus and host's innate and adaptive immunity genes. Persistence tends to be genetically stable and is also highly species-and tissue-specific, demonstrated by the potential devastating effects when a virus jumps from one tissue or species to another, or when the host environment is disrupted [11–13]. Understanding the finer nuances of the persistence of a particular virus in a particular host tissue allows one to understand why a virus wreaks havoc when jumping from one host environment to another and how it affects a cellular pathway in one cell type, but not in another. Persistent functional viruses need to replicate and they need to avoid the immune system; traditionally, these two cell biological aspects have been studied separately, but a more recent example of mammalian herpes virus illustrates how viral interference with growth-promoting and antigen presentation pathways are linked [14–17].

Our close relationship with viruses is illustrated by the number of functional persistent viruses we carry and the persistence of viral remanences in our genomes. Endogenized retroviruses (ERVs) and their defectives—long and short interspersed elements (LINEs, SINEs), long terminal repeats (LTRs), *Alus*—constitute a majority of our ‘junk’ (non-protein coding) DNA [18–20]. The solo LTRs originated from full virus integrations and are present in 330 000 copies, as compared to roughly 23 000 genes [21]. The role of ERVs in the divergence of placental species is commonly accepted: Syncytins that mediate cell to cell fusion and immunosuppression during placenta formation (syncytiotrophoblast) have originated from retroviral *env* genes [22–24]. Each mammalian lineage has syncytins coming from distinct ERVs as a result of independent gene captures occurring separately in the genome of each lineage, making the placenta the most variable organ in mammalian species [24]. Moreover, their expression is also controlled by a complex network of ERVs (mainly their LTRs), derived from distinct retroviral integrations and expressed as non-coding RNAs [24].

Functional viruses that establish a life-long persistence (latency) in humans, such as the gamma herpesviruses Epstein–Barr virus (EBV), human cytomegalovirus (HCMV) and Kaposi's sarcoma-associated herpesvirus (KSHV) are present in the majority of the population and each virus has evolved a balance with a specific host cell type to ensure proliferation and replication without causing too much harm while at the same time preventing detection and destruction by the host's immune system. The EBV infects approximately 95% of the population and its prime resident host cell is the resting B cell [25–27]. HCMV infects 70–100% and establishes latency in CD34^+^ hematopoietic progenitor and myeloid lineage cells [28,29]. The proportion of people infected with KSHV is about 10–40%, with important geographic variations [30,31]. Most herpes virus infections will go unnoticed unless the host's immune system is in an unbalanced state, either via infections like HIV, or via chemically induced immune suppression in connection with, for example, organ transplantation. In such cases, the viruses can cause severe symptoms, including lymphoproliferative disorders and cancers. This illustrates how finely tuned the normal virus–host interactions are and how their cell-transforming capacities are kept in check by the immune system under normal conditions. EBV, for example, is one of the most efficient cell-transforming agents known and exposure of B cells to the virus *in vitro* results in the establishment of immortalized cell lines [32–34]. It is not clear how the balance between the virus's cell-transforming capacity and the immune system has evolved, and whether the immune system actively represses the cell-transforming capacity of the virus or the immortalized cells are actively suppressed. Nevertheless, it points towards a close relationship between the molecular mechanisms of viral immune escape and the regulation of cell growth and proliferation [35,36]. EBV infection prior to adolescence is non-symptomatic, but causes mononucleosis later, with increasingly severe symptoms as we get older. It is interesting that the establishment of persistent infection at a young age does not give symptoms, while many of the acute viruses cause diseases in the young, and it is worth considering that our viral microbiota helps protect from more harmful viral infections, similar to the establishment of the bacterial microbiota. In this respect, it is noteworthy that the SARS-CoV-2 (or COVID-19) gives relatively few symptoms in most younger individuals, suggesting that this virus might become the latest on a long list to form part of our virosphere. Interesting implications of viral persistence to the survival of the host population came from studies on the mouse hepatitis virus (MHV), a murine coronavirus. Mice caught in the wild are persistently colonized with an array of viruses in 80–90% of cases, including MHV, and are perfectly healthy. However, introducing a mouse from the wild to inbred laboratory mice can have a devastating effect as the viruses of the wild mouse interferes with the reproductive biology of the uninfected colony, or may acutely infect pups, depending on the virus strategy, and cause a reproduction collapse. In this case, the persistent version is the winning outcome as it has consequences to the population survival [37,38].

## 2. Viruses and self-identity

The capacity of persistent, defective or functional viruses to protect or to kill cells constitutes a creative and competitive force to understand the survival and evolution of life, and it is interesting to consider how it has evolved. Exchange of information played an essential role in building the particles that eventually could form cells and replicate. These ‘building blocks’ were RNA consortia, later linked with amino acids or other smaller molecules to enhance their identity [39]. Once cells were formed, the exchange of genetic material remained an evolutionary driver, but with the presence of cells, the exchange took the form of virus-like particles with an active capacity to go between cells. As more advanced multicellular organisms evolved, the potential toxic effects of viral infections required a detection system that could prevent some infections while allowing those that were recognized as ‘consortium members’. Interestingly, viruses actively provided the capacity of the host to detect and destroy virus-infected cells, and antiviral defences such as restriction/modification or CRISPR/Cas systems in prokaryotes, RNA interference system (very effective in e.g. *C. elegans*) or innate and adaptive immune response of higher eukaryotes all have viral origins and can be seen as systems enhancing the host identity [4]. Phylogenetic analysis indicates that the most basal members of the TCR genes are the viral *JAM/CTX/PVR* genes, also called the CTX-like family of viral receptors, that support viral interactions and often function as specific virus receptors (PVR, polio virus receptor; JAM, reovirus receptor) [40,41]. Hence, not only our ‘junk’ DNA has a viral origin.

The persistent state of a virus as an ‘addiction module’ was initially discovered in *Escherichia coli* after daughter cells that lost the P1 phage died. The P1 phage produces a stable toxin and a less stable anti-toxin, and thus, cells that lose P1, or for other reasons stop producing the anti-toxin, are killed by the stable toxin using the cell's own programmed cell death systems [42]. If another phage (such as IS2) tries to colonize a P1-carrying cell, it causes an imbalance in toxin/anti-toxin and the cell will die, and thus the concept of addiction acting as an antiviral defence [38,43,44]. The state of one genetic parasite being colonized by another (hyperparasite colonization) is common and affects the relationship of the host with other viruses [44]. The addiction concept to provide resistance to a diverse set of phages and viruses has implications for the colonization of our own microbiota, both in terms of bacteria [45–47] and also of viruses, and has interesting implications for the origin of the viral immune response in higher organisms. A self-destruction mechanism of the infected cells based on a toxin/anti-toxin system (addiction module) is observed in the white spot syndrome virus (WSSV), which is a well-characterized DNA virus causing large crashes in shrimp populations at commercial shrimp farms by producing a pro-apoptotic toxin. In some species, however, the WSSV itself produces an anti-toxin and does not cause any disease symptoms [48,49].

The adaptive immune system first appeared in jawed fish and includes recombination activating genes RAG1 and 2, immunoglobulins, T cells with their receptors (TCRs) and MHC class I and class II molecules [50–53]. There is, however, no living organism representing a gradual acquisition of components of this system, and the rapid occurrence of the adaptive immunity in jawed vertebrates has been attributed to genome duplication due to the colonization of mixed population of viruses (both DNA viruses and retroviruses) and occurrence of endogenous viruses (gypsy-like ERVs) and their defectives (Alus, LINEs, SINEs) [4,54]. In accordance with the virus addiction concept, such a large-scale colonization provides a selective pressure to resist viral competition and an ability to recognize and exclude other virus-infected self cells. Thus, it largely enhanced the self identity of the jawed fish, which now allows recognition of virus-infected self cells. Consequently, both DNA viruses and retroviruses played a role in forming the network of adaptive immune response via providing genes and their regulation, and many of the components of adaptive immunity indeed have a viral-like origin (table 1) [4,54]. For example, the human MHC class I region has 16 distinct human ERVs (HERVs) and the density of the retroelements in this region is 10 times greater than in other regions of the chromosome. These retroelements give rise to a number of non-coding RNAs (ncRNAs) that regulate the expression of various genes, including (but not restricted to) those involved in antigen processing and presentation [58,59,65,66]. One of the most widely studied is long ncRNA HCP5 (*Human Leukocyte Antigen pseudogene P5*), which evolved from an ancient HERV16 insertion (approx. 37 million years ago) and later sequestered the MHC promoter and enhancer region from an ancient *HLA* class I gene [60]. *HCP5* is an antisense retroviral transcript that interacts with regulatory micro RNAs but also binds transcription factors, enhancers and chromatin remodelling enzymes. It is involved in adaptive and innate immune responses and associates with the promotion of some autoimmune diseases and cancers.

**Table 1.** Viral impact on the origin and evolution of immune system.

components of innate and adaptive immune response	relation to viruses	Ref.
interferon gamma network	binding sites for IFN gamma-regulated transcription factors STAT1 and IRF1 are composed of LTR regions from endogenized gammaretrovirus family MER41 that invaded the genome of an anthropoid primate ancestor approximately 45–60 million years ago	[55]
immunoglobulin (Ig) receptors [T-cell receptors (TCRs), B-cell receptors]	the basic structure of Ig receptors is found within CTX (*cortical thymocyte marker of Xenopus*)-like family of viral receptors including junctional adhesion molecule (JAM), poliovirus receptor (PVR), coxsackie and adenovirus 5 receptor (CAR), signalling lymphocytes activation markers (SLAM)	[40,56]
RAG1/2 system	it acts like ‘cut and paste’ transposon—the most basal version is that of phage Mu; it also shows similarity to the RNAse H fold of retroviral integrase	[54,57]
MHC region	human MHC region is particularly dense with viral-derived elements (ERVs, Alu, SINEs, LINEs) which contribute to its evolution as e.g. recombination sites for duplication; they give rise to numerous regulatory ncRNAs (miRNAs, lncRNAs) that regulate expression of various genes	[58–64]

## 3. The major histocompatibility class I pathway and viral interference

**Figure 1.**
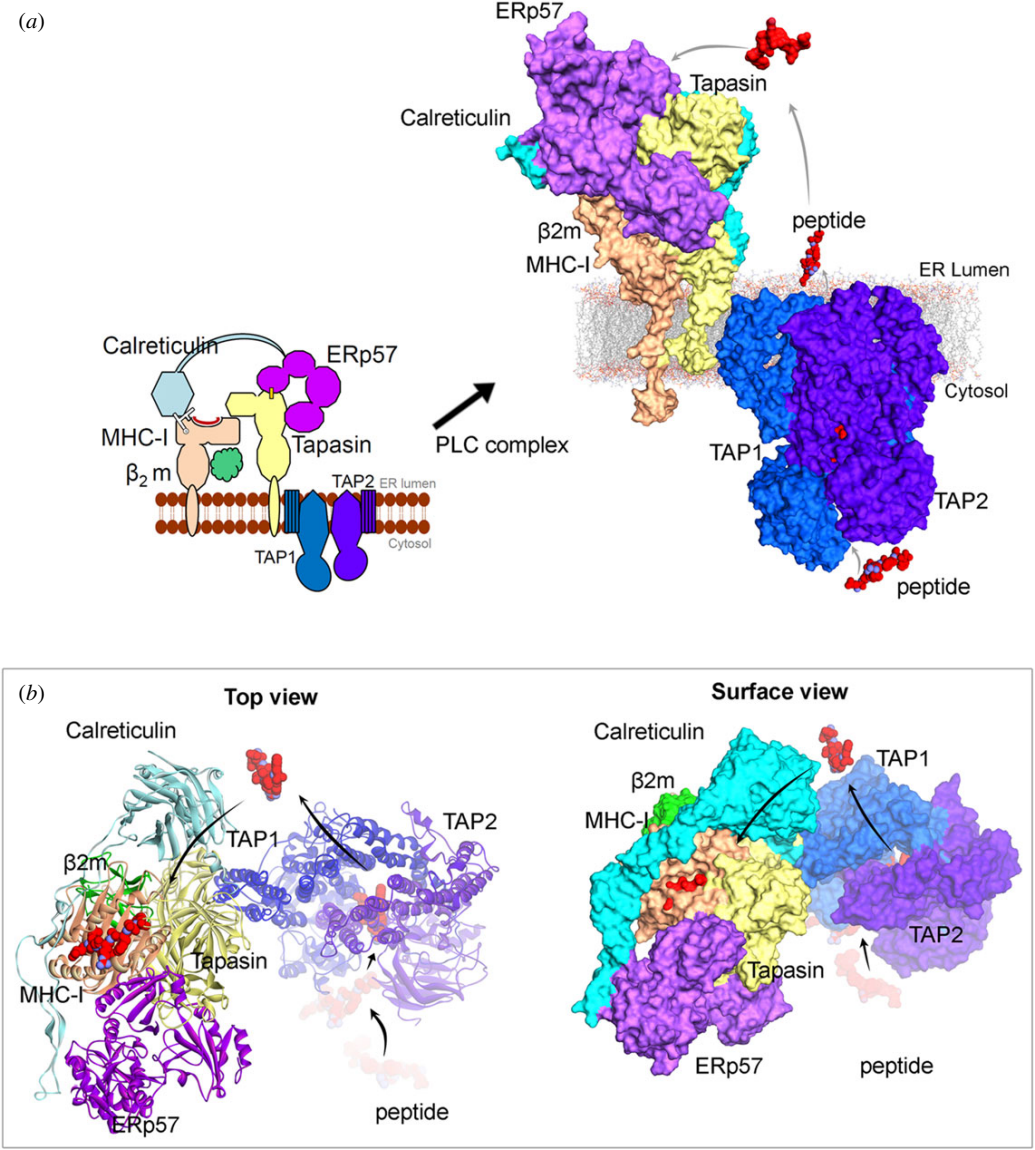
Different components from the peptide-loading complex (PLC) involved in the antigen presentation. (*a*) Representation of proteins MHC I, *β*2m, Calreticulin, ERp57, Tapasin, TAP1 and TAP2 and their possible protein–protein interfaces. Right panel describes the possible way of transporting peptide from cytosol-ER lumen, that could be presenting over MHC-I molecules. (*b*) The top surface view of the PLC, highlighting the peptide-binding cavity from MHC I molecule in the presence of a peptide bound to it. For (*a*) and (*b*), the template structures used to model PLC were PDB: 6eny [67] (MHC-I, *β*2m, Calreticulin, ERp57 and Tapasin) and PDB: 5u1d [68,69](TAP1 and TAP2). The three-dimensional view of the structures were prepared using BIOVIA Discovery Studio (Dassault Systèmes, BIOVIA Corp., San Diego, CA, USA).

Peptide substrates of approximately 30 amino acids or longer need a proteasomal activity to be presented by MHC class I molecules. The TAP peptide transporter consists of TAP1 and TAP2, and is located in the membrane of the endoplasmic reticulum (ER) and brings peptides into the ER where they are further trimmed by the ERAPs1–2 aminopeptidases to 8–10 amino acid lengths before introduced to the peptide-loading complex (PLC). The PLC is linked to the TAP and includes the MHC class I molecule, B2-microglobulin, Tapasin and Erp57 that helps stabilize the complex before peptide loading and export to the cell surface via the Golgi (figure 1). Most persistent viruses have been shown to interfere with the MHC class I pathway, and perhaps this holds true for all viruses of this category. Different viruses target different components of the TAP-PLC complex. This might seem surprising and one could argue that if a virus found a clever way to interfere with a specific component of the TAP-PLC to bypass the MHC class I pathway, this would be copied by them all. However, if one sees the TAP-PLC as one functional unit, then this is indeed the case.

Herpes simplex (HSV) factor ICP47, the US6 of the HCMV as well as the BNLF2 of the EBV all target TAP. Interestingly, though patients with TAP deficiency syndrome do suffer from recurrent bacterial infections of the upper respiratory tract manifesting within the first 6 years of life, severe viral infections are noticeably absent and normal antibody titres against several viruses have been demonstrated in most patients [70–72]. This can perhaps be explained by more recent observations showing that viral peptides are presented to the class I pathway in a TAP-independent fashion [73–76]. Furthermore, fusing the bacterial multidrug resistance proteins A and B (TmrAB) transporter to the fragment of the transmembrane domain of TAP2 mediating interaction with tapasin restores antigen presentation in cells lacking TAP, indicating that a major role of TAP in antigen presentation is via its role in regulating PLC dynamics [77].

A question regarding peptide transport is what happens to peptides that are not loaded on the class I molecules. There is not much selection for which peptides that are transported into the ER by the TAP except for a few charged residues in the N- and C-termini. Considering the vast amount of peptide products produced by the proteasome every minute and the relative few MHC class I molecules available for peptide loading, only a very small amount of peptides can be bound to the class I molecules [78,79]. What happens to the rest? The ER is an environment sensitive to the presence of unfolded proteins and when these interact with the BiP chaperone, it triggers the unfolded protein response [80]. If antigenic peptide substrates are too small to interact with BiP, or if the charged N- and C-termini of antigenic peptides substrates is sufficient to prevent BiP recognition and is not known. Misfolded proteins are retrotranslocated out to the cytoplasm for degradation in a ubiquitin-dependent fashion by the ER-associated degradation (ERAD) system [81–83]. It is unlikely that this pathway also expels peptide substrates for the MHC pathway, and it has not been observed that such are ubiquitinated. But as the ER membrane does not allow passive transport of peptides either, it leaves a gap in our understanding of how the majority of peptides substrates that enter the ER via the TAP are taken care of.

## 4. Viral and cancer immune evasion

**Figure 2.**
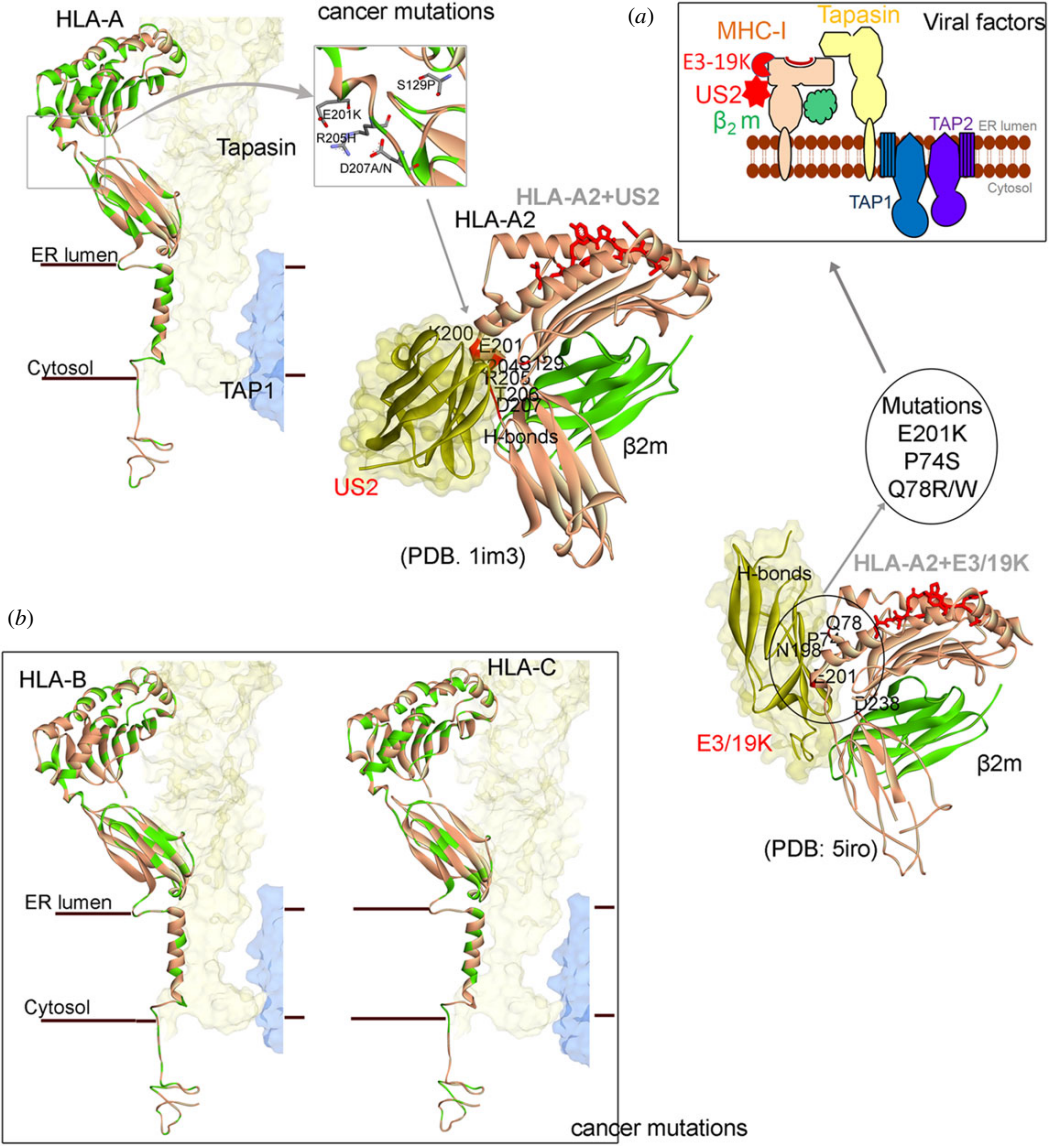
Viral protein binding with different regions of MHC I molecules compared with the amino acid variants (retrieved from cBioPortal [86]) from different cancer types. (*a*) Mutations in HLA-A from different cancer types (mutations are marked in green colour), compared with the viral interaction surface with HLA-A2. The crystal structure of US2 with HLA-A2 (PDB: 1im3 [87]) and E3/19 K with HLA-A2 (PDB: 5iro [88,89]). Hydrogen bond interactions between the viral proteins US2, US3 and E3/19 K with HLA-A2 are marked as red on the protein structure. (*b*) Cancer mutations marked with green colour on the HLA-B and HLA-C. Colour scheme: carbon in grey, nitrogen in blue and oxygen in red. The layover of cancer mutations on structure, and H-bond interactions were identified using BIOVIA Discovery Studio (DassaultSystèmes, BIOVIA Corp., San Diego, CA, USA).

**Figure 3.**
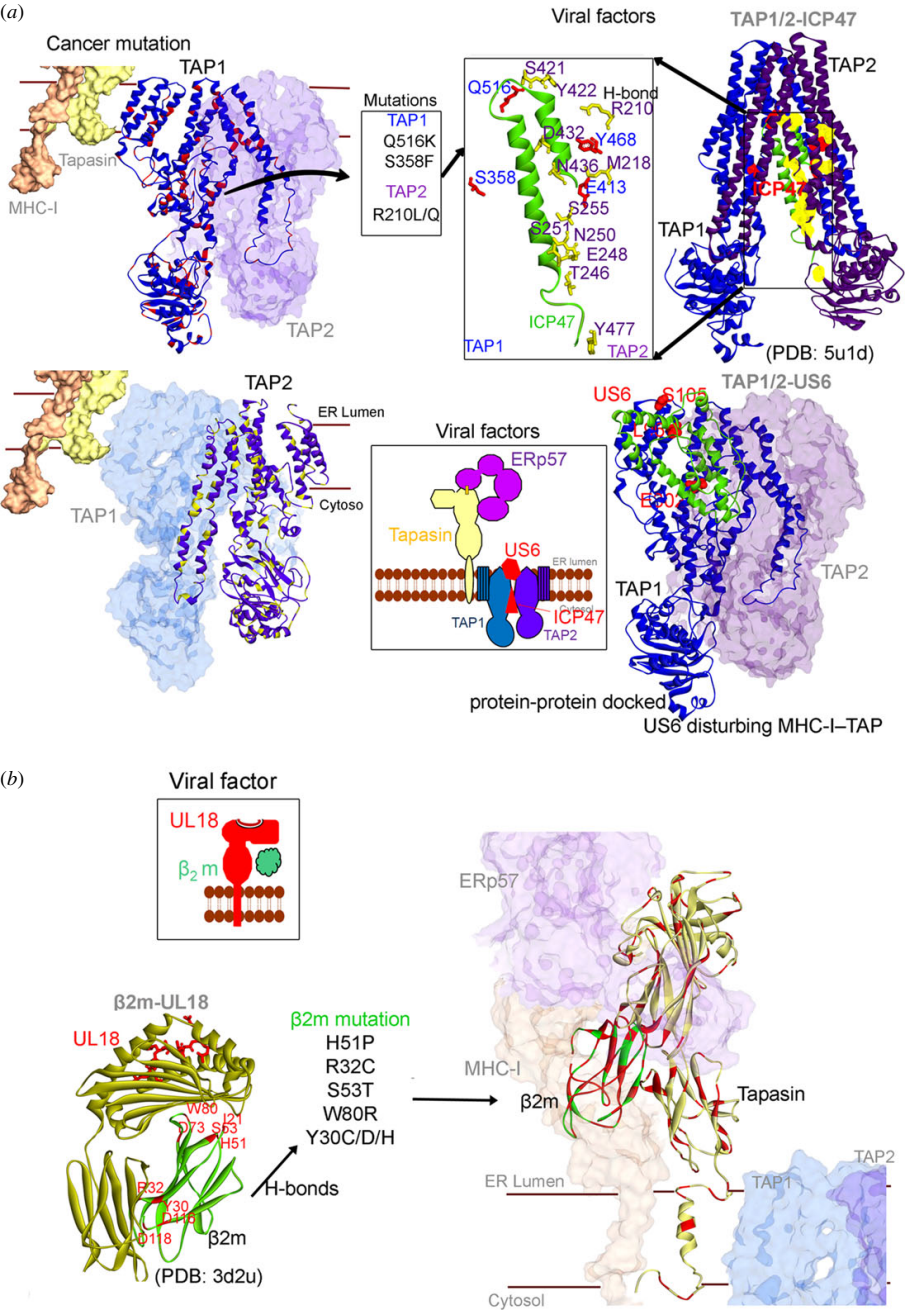
Comparison of cancer mutations (from cBioPortal [86]) with viral proteins interacting with the peptide-loading components. (*a*) TAP1 and TAP2 cancer mutations, presented as red and yellow, respectively. The viral protein ICP47 crystal structure with TAP1 and TAP2 (PDB; 5u1d [69]); residues involved in H-bond interaction with ICP47 from TAP1 and TAP2 are presented as sticks with labelled colour blue and violet, respectively. The viral protein US6 is suggested to bind the TAP1 and TAP2 blocking the peptide transport process [90]. For the US6 viral factor, its structure was modelled using homology modelling approach (swiss-model [91]) and the protein–protein docking was performed with TAP1 (using ZDOCK [92]), resulting US6-TAP1/TAP2 conformation correlates with the data available in the literature [90,93,94]. H-bond formed between US6 and TAP1 is marked in red colour. (*b*) The *β*2m and Tapasin protein cancer mutations marked in red colour. Viral protein UL18 mimicking human MHC I and interacting with *β*2m (PDB: 3d2u [95]) is shown, residues involved in H-bond are marked in red colour and compared with the cancer mutations. The layover of cancer mutations on structure and H-bond interactions were identified using BIOVIA Discovery Studio (DassaultSystèmes, BIOVIA Corp., San Diego, CA, USA).

In addition to detecting and destroying virus-carrying cells, our immune system also serves to eliminate damaged or harmful cells. In both viral and cancer immune evasion, the expression of neoantigens on the MHC-I molecules plays a key role, and it could therefore be expected that the targets for viral and cancer immune evasion are similar. Indeed, downregulation of MHC-I molecules is frequent in cancers and also a common viral target. The discovery of transmembrane E3 RING-CH-type ubiquitin ligases (MARCH) came from studies on the Kaposi's sarcoma viral factors kK3 and kK5 and the equivalent cellular factors were discovered later. It is, however, not clear if a virus gave the cells MARCH, or if viruses acquired MARCH from the host. The MARCH proteins (MARCH1–10) are regulated by inflammatory signals and play important roles in various aspects of the immune system including DC differentiation. They control MHC-I and MHC-II internalization via monoubiquitination, or the degradation via lysosomal or proteasomal pathways following polyubiquitination. kK3 and kK5 targets MHC on the cell surface, whereas the MHV86 mK3 sits on the TAP molecules and wait for the nascent MHC-I to enter the ER and promotes ubiquitination and promotes ERAD. The US2 and US11 also target MHC-I for ERAD but via a slightly different mechanism. They cause retrograde transport, or dislocation, of newly synthesized HLA-I heavy chains by hijacking the cellular quality control pathway ERAD, providing it with folding-competent MHC-I to induce its rapid proteasomal degradation [84,85]. MARCH proteins are dysregulated in various cancers, suggesting that viruses and cancers share common strategies in exploiting ubiquitination pathways for immune evasion. A closer examination of the interfaces between viral interacting proteins and cancer mutations (figures 2 and 3) further illustrate common viral and cancer strategies. The US2 binds HLA-A2 (MHC I) at the junction of the peptide interactive groove and on the MHC I surface, which allows US2 to bind independently of peptide sequence [87]. The human adenovirus E3/19 K immunomodulatory protein retains HLA-A and HLA-B, but not HLA-C, within the ER [88]. The human cytomegalovirus US3 binds to MHC-I in the ER and interferes with tapasin-dependent peptide loading [96–99]. Moreover, the molluscum contagiosum virus protein MC80 binds to tapasin, leading to its degradation and the loss of TAP proteins [100]. The TAP complex is targeted by several viral factors and the ICP47 (Infected Cell Protein 47) from the herpes simplex virus locks TAP in an inactive conformation that prevents its role in the PLC [69,90,93,94]. Similar, the HCMV-encoded US6 glycoprotein interacts with the TAP1 subunit and blocks peptide entry into the ER. Furthermore, the ATP-driven conformational change of TAP1 required for peptide transport from cytosol to ER is the target of the US2 [93]. Peptide transport is energy-dependent, and TAP ATP binding and hydrolysis is a target for the cowpox CPXV012 protein [101]. BNLF2a from Epstein–Barr virus subsequently arrests TAP in a translocation-incompetent conformation [94,102].

Using the cancer genome atlas (TCGA) and the cBioPortal database to trace cancer mutations in different HLA types (HLA-A, HLA-B, and HLA-C; figures 1 and 2) suggests that even though there are similarities between HLA types, several mutations occur only in a particular HLA molecule and in the *α*3 domain and the transmembrane domains [13]. Furthermore, the HLA-A molecules have overall more residues mutated as compared to HLA-B and -C. HLA-C instead shows more mutations in the peptide-binding groove (figure 2*b*), and it is interesting to note that HLA-C is the major inhibitory ligand for killer immunoglobulin-like receptors (KIRs), which might help to explain why viruses and cancers target this haplotype differently. HLA-C2 is more inhibiting to NK cell activation as compared to HLA-C1 and the E3/19 K does not interact with HLA-C [88]. The TAP1/2 and *β*2m proteins are frequently mutated in different cancer types (figure 3). In addition, analysing the variations in Tapasin suggests that the Ig-like domain and the c-terminus region are heavily mutated. It is known that the scoop loop is of critical importance for TAPBPR-mediated stabilization of empty MHC-I client molecules [103,104], which is also found mutated in different cancer types.

A majority of viral proteins interact with regions in the PLC which are highly mutated in cancers. For example, the viral factors US2 [87] and E3/19 K [88] form hydrogen bond interactions with the HLA-A2 residues (figure 2) that are mutated in different cancer types. Similarly, UL18 viral factors bind to *β*2m residues that are mostly mutated in cancer types (figure 3*b*). In particular, looking at the TAP transporters from the PLC complex, the viral protein ICP47 forms h-bond interactions with four amino acids (S358, E413, Y468 and Q516) of TAP1 protein, from which two residues (S358F and Q516 K) were mutated in cancer (figure 3*a*). Conversely, TAP2 protein has formed more interactions (R210, M218, T246, E248, N250, S251, S255, N436, D432, Y422, S421 and Y477) with ICP47 compared to TAP1, and only one residue (R210 L/Q) is found mutated in cancer (figure 3). With a glance from these observations of the viral proteins (US2, E3/19 K, ICP47 and UL18) and the cancer mutations, it could be suggested that they share a common interface to interfere with the PLC (figures 2 and 3).

## 5. Impact of viral infections on immune checkpoints

Recent years have seen a rapid expansion in immune-based cancer treatments, most notably using antibodies that target immune checkpoint (IC) receptors PD-1 or the co-regulatory inhibitory cytotoxic T cell receptor CTLA-4, and their ligands PD-L1/2 and CD86 and CD80, respectively. The PD-1 is expressed mainly on T- and B-lymphocytes as well as NK cells and monocytes, whereas the PD-L1 is expressed in most tissues, including cancer cells. PD-L2 is less abundant and its role is still not completely understood [105,106]. Upon engagement by its ligand, PD-1 acts as a break to T cell cytotoxic activity and PD-1/PD-L1 and cancer cells employ different approaches to exploit this to evade the immune system. The importance of this approach for cancer immune evasion is illustrated by the use of various antibodies that block these interactions for the treatment of various cancers. Considering the importance of the ICs in cancer cell immune evasion, it would be expected that viruses would also target the immune checkpoints. However, the cellular response to acute viral infection is aimed at driving the expression of the PD-1/PD-L1 axis, perhaps to act as a rheostat to regulate the strength and duration of the immune response [107]. A direct interference with PD-1/PD-L1 axis by viral antigens has not been reported but *Herpesviridae* [108] produces a protein that mimics IL-10 and further increases PD-1/PD-L1 signalling on immune cells [109]. Furthermore, Kakizaki *et al.* [110] observed increased PD-L1 expression on monocytes that was induced by extracellular vesicles (EVs) released by HBV infected hepatocytes [110]. Prolonged and persistent PD-1/PD-L1 stimulation observed in cancer, and persistent viral infections such as the LCMV and HBV, ultimately lead to T-cell functional exhaustion and reflect a state of adapting the T cell response to chronic inflammation. Studies in a murine model for HCV (Norway rat Hepacivirus) indicated that PD-1/PD-L1 blockade implemented at the early stage of the infection indeed reduced viral load, but no significant improvement was reported as the chronic infection progressed [111]. Consequently, ICs might pose a difficult target for viruses. We do not fully understand how the IC functions and why it is not a suitable viral target.

## 6. The source of antigenic peptides: from immune evasion to cell cycle control

The first step in antigen presentation is, in fact, not proteasome-mediated degradation of the peptide substrate that is commonly pictured but rather the synthesis of the antigenic peptide substrate [112–114]. The different viral factors mentioned that interfere with the PLC-TAP all act in *trans*, and hence a certain amount of viral factors have to be produced in order to have an immune evasive effect. But what protects the host cell from the immune system before a sufficient level of trans-acting factors has been produced? To fill this gap in their protective repertoire, viruses can also target the production of antigenic peptide substrates [115–117].

Full-length proteins are, in fact, poor substrates for the class I pathway, while non-canonical sources are presented. For example, antigenic peptide substrates can be initiated from leucine codons in the 3′ UTR as well as from pre-spliced mRNAs [118,119]. The nature of the pre-spliced mRNA translation event is still not clear, but it can be distinguished from canonical translation [16,113]. It is not known if such pioneer translation products (PTPs) derived from pre-spliced mRNAs have other functions in the cell apart from an antigenic peptide source but it is plausible that the MHC class I pathway has its ‘own’ mRNA translation event [120]. The rationale for having alternative translation products rather than full-length proteins as a source of peptide substrates relates to the limited amount of MHC class I molecules as compared with the enormous amount of protein-derived degradation products derived from full-length proteins and the need for the immune system to stay sensitive to viral antigens [120–123]. Hence, if all peptide products derived from proteasomal degradation of full-length proteins had access to the class I pathway, it would be a lottery whether non-self antigens were to be presented or not. Furthermore, if the first peptide products produced from a viral mRNA are selected for the MHC class I pathway, it ensures a rapid detection of viral infection before viral *trans*-acting factors have a chance to interfere with the class I pathway. An alternative and perhaps specific translation event for the production of antigenic peptide substrates for the MHC class I pathway might seem excessive, but if one considers the fundamental importance of the immune system in evolution, it puts things in a different perspective, and a specific translation event to help detect and eliminating virus-infected cells is perhaps not such an extreme possibility. It is possible that this non-canonical translation event is a reminiscence of a co-transcriptional translation event, similar to the prokaryotic, that became dedicated to producing antigenic peptides for the MHC class I pathway, while the canonical translation machinery evolved with its well-defined and regulated protein synthesis.

One would expect that viruses would have a strategy to counteract an alternative source of antigenic peptides and it appears that one approach is to minimize the translation of the viral mRNAs using cis-acting mechanisms [124]. This is the case for the EBNA1 of the EBV, which is the only viral antigen expressed in resting B cells and in EBV-carrying Burkitt's lymphomas, and compounds that stimulates EBNA1 translation also increase the production of antigenic peptides from the EBNA1 message [124]. The LANA1 of the KSHV is using a similar *cis*-acting mechanisms but it is not yet known if other viruses are also using a similar strategy [116,125,126].

Apart from evading the immune system, latent viruses can also control the proliferation of the host cell. Despite being two essential aspects for viruses, there are yet few examples where these two concepts are shown to be interlinked. Simian, adeno and human papilloma viruses express proteins that interact with the retinoblastoma protein (pRb) and the p53 tumour suppressors [127–129]. The interaction with pRb by E6, E1A or large T antigen releases the E2F1 transcription factors and promotes cell growth and proliferation via a number of downstream target genes including cyclins and c-myc [130,131]. The latent herpes viruses do not seem to express proteins that bind pRb, but more recent works on EBV suggest that the virus is using another mechanism to induce E2F1 expression [14]. Two EBNA1 transgenic animal models show an inverse correlation with tumour phenotype and EBNA1 expression [132,133]. Thus, the less EBNA1 expression, the more tumours, which is counterintuitive, as it would be expected that more of an oncogene would result in more tumours. It turns out that it is not the EBNA1 protein that is oncogenic—it is the *EBNA1* mRNA. Under conditions when EBNA1 suppresses its own mRNA translation it activates E2F1 synthesis. The signalling pathway mediating this link requires the PI3 K*δ*, and inhibition of PI3 K*δ* prevents the expression of E2F1 and its downstream target c-myc, and suppresses growth of EBNA1-induced tumour cells [14]. Hence, by suppressing its own synthesis, EBNA1 evades MHC class I antigen presentation, while at the same time, targeting the pRb tumour suppressor pathways. This example illustrates how two key viral targets for a successful latent virus–host interaction have co-evolved.

## 7. Non-protein-mediated control of the host cell

As the immune system uses peptides to distinguish between self and non-self it raises the question to what extent viruses use non-coding RNAs to manipulate the host cell environment to minimize the production of antigenic peptides and acquire stable infection. All human DNA viruses use ncRNAs that interact with nucleotides and proteins, and regulate expression of both cellular and viral genes (both individual genes and gene sets) via both induction and interference with gene expression, serving as scaffolds to ribonucleoproteins (RNPs) or as guides in target recognition within the genome. First reports on the virus expressing miRNAs came from studies on EBV in 2004 [134], and to date 44 EBV-encoded miRNAs produced have been described [135]. EBV miRNAs downregulate the viral genes such as the viral DNA polymerase *BALF5*, and the early EBV genes *BZLF1* and *BRLF1* to keep the viral levels in check and to prevent the transition to the lytic state [136]. Viral miRNAs also interfere with the host immunity by targeting pattern recognition receptors such as RIG-I (*retinoic acid-inducible protein 1*) [137] and IFN gamma-STAT1 pathway [138] thus suppressing interferon signalling and the antiviral response. They also downregulate cytokines and chemokines production and block the antigen presentation and subsequent T cell responses. They are also engaged in the control of cell proliferation and apoptosis by targeting pro-apoptotic genes such as *BAD, BIM, BAX, MAP3K5, PUMA* and capase-3 for inhibition of apoptosis.

A different aspect of using RNAs to manipulate the host comes from the concept of quasispecies that are diverse RNA species derived from RNA viruses due to RNA polymerase infidelity. These can form groups of interactive variants that are crucial for the viral population to attain fitness and to persist in the host. This interesting concept was initially thought of as an error-based selection of the fittest (master) type replicator from the spectrum of mutants. More recent models indicate that quasispecies is a result of a collective viral evolution and not as separate species that together contribute to the characteristics of the population [139–141]. For example, in poliovirus pathogenesis, the generation of diversity (not errors) and cooperation between quasispecies is key to attain disease-associated viral fitness, and a virus carrying a high-fidelity polymerase generates less genomic diversity and is less capable to adapt to adverse growth conditions [142]. HCV quasispecies form swarms of variants that both complement and interfere with the replication of the whole population. Preclusions between different HCV quasispecies populations (clades) have been observed *in vivo* after blood donation and liver transplants of HCV-infected donors to the HCV-infected recipients [143–145]. Only one HCV strain would survive the mixing of quasispecies clades, indicating that the selection occurs on the population level. The joining of RNA species to form a collective occurs via RNA stem-loops structure interactions. Thus, RNA stem-loop structures provide molecular identity to the RNA species, and individual membership is therefore dynamic, but must be coherent with the group [146].

## 8. Conclusion

Viruses skilfully evade both innate and acquired immune responses by targeting factors within pathways distinguishing self from non-self, but at the same time viruses have contributed to mechanisms whereby cells sense viral infections. This apparent contradiction warrants a deeper look at virus–host interactions, what each partner is trying to achieve and why in some cases they coexist in harmony but in others they do not. Viruses are the most abundant entities on the planet and all species live in their corresponding virosphere. While viruses both cooperate and compete to infect their respective hosts, they also provide ready network solutions in order to enhance the host's self identity. An exchange of genetic material between the two, in case of persistent viruses, has evolved to become a fine-tuned balance of coexistence dating back to the origin of that host. And when the immune system is altered, or when the virus jumps species or cell type, the consequences can be severe. The high number of epigenomic viruses and the accumulation of virus-derived information in the ‘non-coding DNA’, as well as in the genes, and the role of these elements in regulating network functions is hard evidence of this evolutionary complexity.

Viral persistence can be seen as consequential to the survival of the colonized population, and P1-infected *E. coli* or wild mouse carrying persistent MHV are examples where viruses outcompete the noncolonized bacteria or mice. The addiction model in which viruses have different host- and cell-type-specific mechanisms to set the persistence helps in better understanding the antiviral immunity processes, their origin and what happens when we are targeted by viruses we are not used to. The connection of these mechanisms to cell proliferation and differentiation underlies the long-term coevolution of human and these viruses.

## Data accessibility

For preparing the figures and representations, we have used the crystal structures that are already available in the PDB database (https://www.rcsb.org/), and for each protein structure presented in this manuscript, we have provided the unique PDB ID from the PDB database.
